# Cryo-EM structure of the SEA complex

**DOI:** 10.1038/s41586-022-05370-0

**Published:** 2022-10-26

**Authors:** Lucas Tafur, Kerstin Hinterndorfer, Caroline Gabus, Chiara Lamanna, Ariane Bergmann, Yashar Sadian, Farzad Hamdi, Fotis L. Kyrilis, Panagiotis L. Kastritis, Robbie Loewith

**Affiliations:** 1 Department of Molecular and Cellular Biology, University of Geneva, Geneva, Switzerland; 2 CryoGEnic facility (DCI Geneva), University of Geneva, Geneva, Switzerland; 3 Interdisciplinary Research Center HALOmem & Institute of Biochemistry and Biotechnology, Martin Luther University Halle-Wittenberg, Halle, Germany; 4 Swiss National Centre for Competence in Research (NCCR) in Chemical Biology, University of Geneva, Geneva, Switzerland

**Keywords:** Cryoelectron microscopy, TOR signalling, Cryoelectron microscopy, Nutrient signalling

## Abstract

The SEA complex (SEAC) is a growth regulator that acts as a GTPase-activating protein (GAP) towards Gtr1, a Rag GTPase that relays nutrient status to the Target of Rapamycin Complex 1 (TORC1) in yeast^1^. Functionally, the SEAC has been divided into two subcomplexes: SEACIT, which has GAP activity and inhibits TORC1, and SEACAT, which regulates SEACIT^2^. This system is conserved in mammals: the GATOR complex, consisting of GATOR1 (SEACIT) and GATOR2 (SEACAT), transmits amino acid^3^ and glucose^4^ signals to mTORC1. Despite its importance, the structure of SEAC/GATOR, and thus molecular understanding of its function, is lacking. Here, we solve the cryo-EM structure of the native eight-subunit SEAC. The SEAC has a modular structure in which a COPII-like cage corresponding to SEACAT binds two flexible wings, which correspond to SEACIT. The wings are tethered to the core via Sea3, which forms part of both modules. The GAP mechanism of GATOR1 is conserved in SEACIT, and GAP activity is unaffected by SEACAT in vitro. In vivo, the wings are essential for recruitment of the SEAC to the vacuole, primarily via the EGO complex. Our results indicate that rather than being a direct inhibitor of SEACIT, SEACAT acts as a scaffold for the binding of TORC1 regulators.

## Abstract (web summary)

The cryo-EM structure of the yeast SEA complex suggests that SEACAT functions as a scaffold for binding TORC1 regulators.

## Main

In the budding yeast *Saccharomyces cerevisiae*, the Target of Rapamycin Complex 1 (TORC1) resides on the vacuolar membrane and receives nutrient-derived inputs from the EGO complex (EGOC)^5^, the yeast counterpart of the mammalian Ragulator-Rag complex^6^. Gtr1 and Gtr2, the two Rag-family GTPases found in the EGOC, regulate TORC1 activity depending on their nucleotide-loading status. Gtr1 and Gtr2 are regulated by dedicated GAPs, the Seh1-associated complex (SEAC)^1^ and Lst4/Lst7 (ref. ^7^), respectively. The SEA complex (SEAC) was initially described as a coatomer-related complex^8^ and was later reported to be functionally divided into two subcomplexes: SEACIT, which possesses Gtr1 GAP activity and thus inhibits TORC1 (ref. ^1^), and SEACAT, which ostensibly antagonizes SEACIT and thus activates TORC1 (ref. ^2^). In mammals, the SEAC counterpart (GATOR) is also functionally divided into a GAP subcomplex (GATOR1) and its regulator (GATOR2)^3^.

The SEAC is composed of eight subunits^8^ (GATOR subunits in parentheses^3^): Sea1/Iml1 (DEPDC5), Npr2 (Nprl2) and Npr3 (Nprl3), which form the SEACIT (GATOR1) subcomplex, and Sea2 (Wdr24), Sea3 (Wdr59), Sea4 (Mios), Seh1 (Seh1L) and Sec13 (Sec13), which form the SEACAT (GATOR2) subcomplex. In GATOR1, Nprl2 contains the catalytic ‘arginine finger’ (Nprl2^Arg78^)^9^, whereas in yeast, this function was assigned to an arginine in Sea1 (Sea1^Arg943^)^1^. In addition, Ragulator-Rag can bind GATOR1 in ‘inhibitory’ and ‘GAP’ modes^10^. Presently, it is unclear whether the GAP mechanism and binding modes observed in GATOR1 are conserved in SEACIT.

Despite reports that mammalian nitrogen sensors regulate GATOR1 activity via binding to GATOR2 (refs. ^11–13^), how GATOR2 regulates GATOR1 is unknown, and the lack of structures has precluded a better understanding of this pivotal axis of TOR signalling. Hence, we set out to solve the structure of the homologous SEAC.

## Cryo-EM structure of the SEAC

**Fig. 1:**
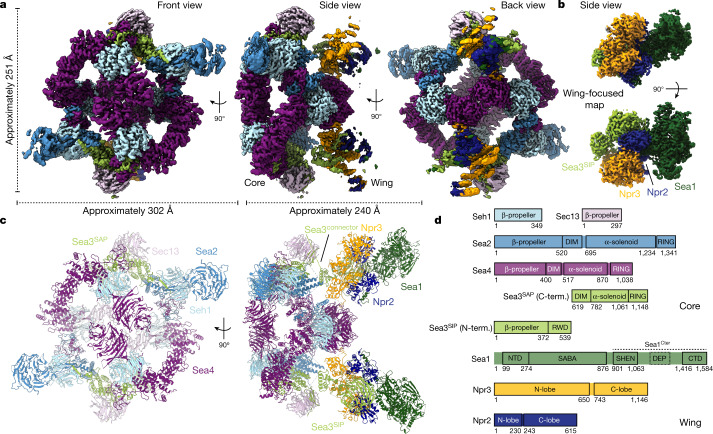
Cryo-EM structure of the SEAC. **a**, Consensus cryo-EM map shown in front, side and back views. **b**, Focused map on the wing module. **c**, Model of the eight-subunit SEAC. **d**, Domain organization of SEAC subunits. N-term., N-terminal; C-term., C-terminal.

**Extended Data Fig. 1:**
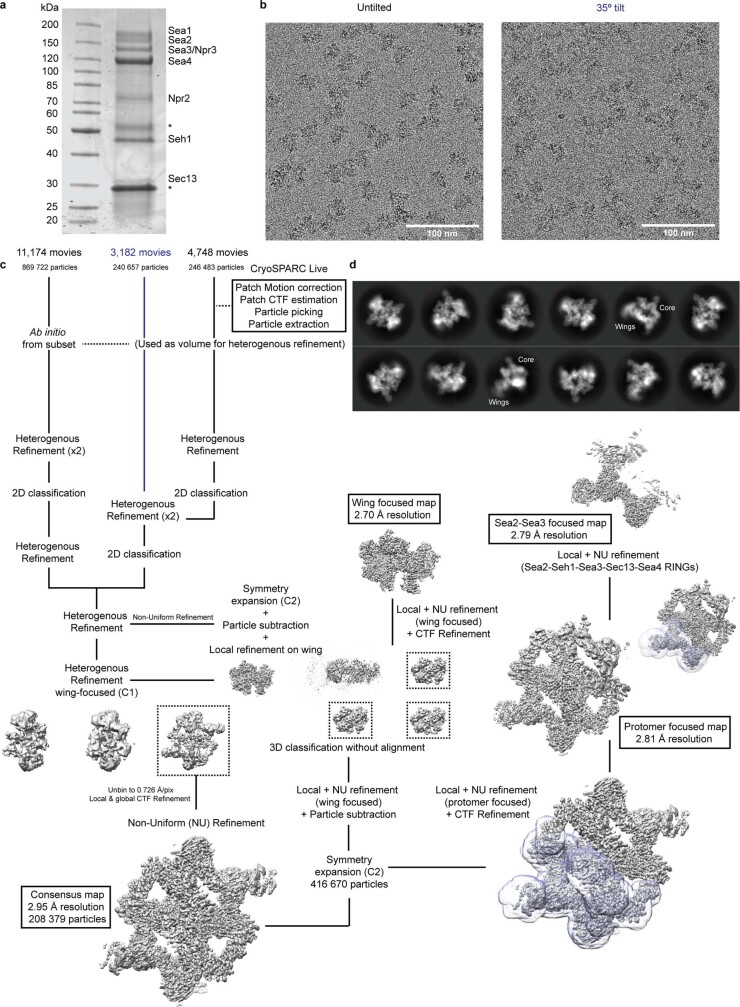
Cryo-EM structure determination of the SEAC. **a**, Coomassie stained SDS-PAGE of purified SEAC. Asterisks indicate presence of IgG and TEV protease in the eluted sample. For gel source data, see Supplementary Fig. 1. **b**, Representative micrographs for untilted and tilted (blue) datasets. **c**, Cryo-EM processing pipeline. Focused masks are shown on top of the 3D volumes. **d**, Representative 2D class averages from the final subset of particles used in the reconstruction. Core and wing modules are indicated in two class averages.

**Extended Data Fig. 2:**
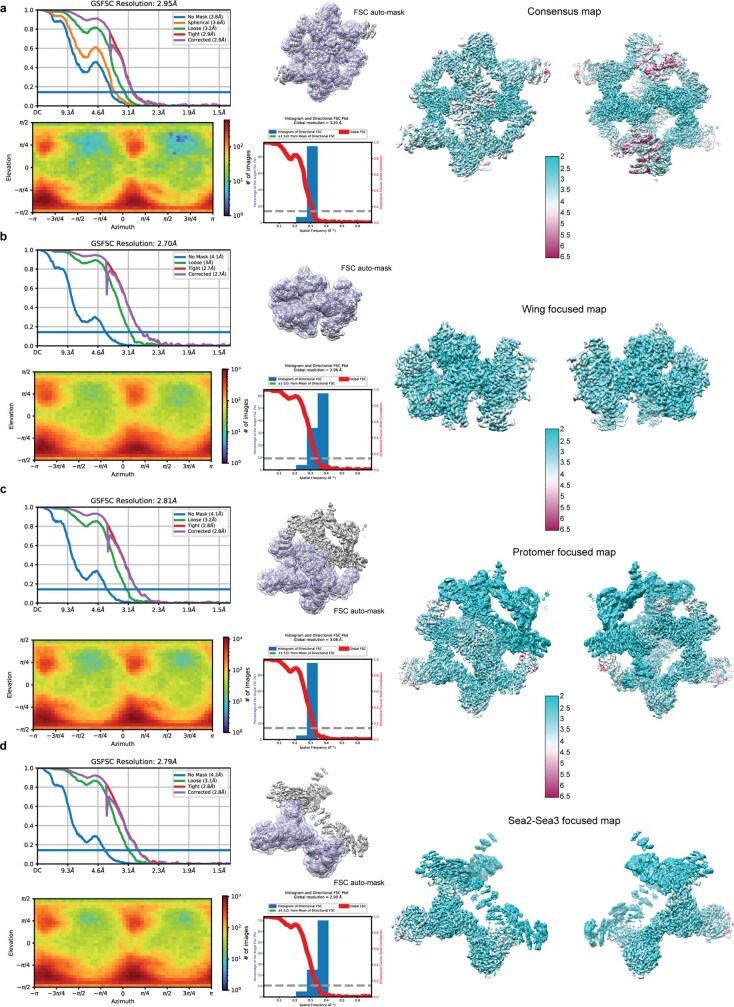
Resolution of cryo-EM reconstructions. **a-d**, Fourier shell correlation (FSC) plots between half-maps, mask used for determination of the average resolution at FSC 0.143, orientation distribution plot, directional (3D FSC) and local resolution estimates for the consensus map (**a**), wing focused map (**b**), protomer focused map (**c**) and Sea2-Sea3 focused map (**d**).

**Extended Data Fig. 3:**
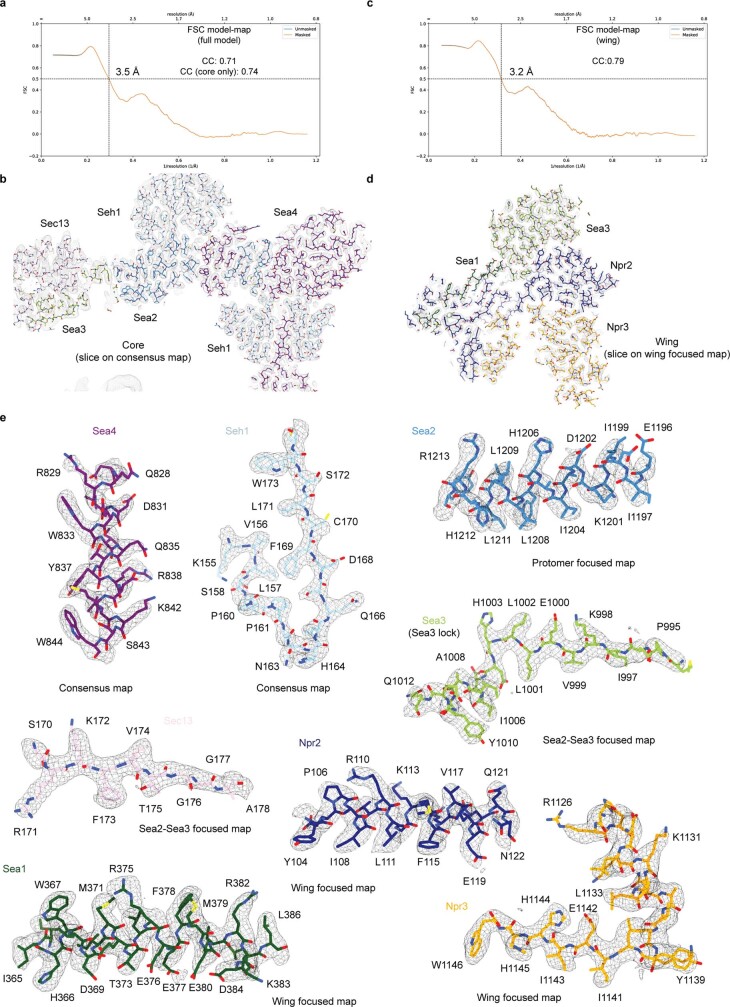
Model to map fit. **a**, FSC between model and map for the full complex against the consensus cryo-EM map. **b**, Model fit to the consensus map. **c**, FSC between model and map for the wing against the wing focused cryo-EM map. The cross-correlation (CC) between model and maps is indicated. **d**, Model fit to the wing cryo-EM map. **e**, Representative cryo-EM densities fitted to the model.

The endogenous SEAC was purified from yeast cells using a gentle protocol, yielding an intact and relatively stable complex (Extended Data Fig. 1a). After extensive sample and data collection optimization, a cryogenic electron microscopy (cryo-EM) reconstruction of the SEAC at an average resolution of 3.0 Å was attained (Fig. 1a and Extended Data Fig. 1b–d). The structure is composed of a dimeric central core that serves as a symmetrical binding platform for two identical flexible wings. We used a series of masks and focused refinements to improve the resolution (Extended Data Figs. 1 and 2), yielding maps at 2.8 Å and 2.7 Å resolution for the core and wings, respectively (Fig. 1b). Using AlphaFold structure predictions^14^ and crystal structures of Seh1 and Sec13, we built a model of the octameric SEAC (Supplementary Table 1 and Extended Data Fig. 3) that is consistent with previous cross-linking and proteolysis data^15^, and which explains previous biochemical observations (see below).

The SEAC forms a hollow, *C*2-symmetric cage-like structure with dimensions of approximately 251 Å × 302 Å × 240 Å, containing 22 protein chains with a mass of approximately 2 MDa (Fig. 1c). There are two copies of each subunit, except for Sea4 and Seh1, which are present in four and six copies, respectively. The central core contains Seh1, Sec13, Sea2, Sea4 and Sea3^Cter^, whereas the wings comprise Sea3^Nter^, Sea1, Npr2 and Npr3 (Fig. 1d). Consistent with functional studies, core subunits have been previously assigned to SEACAT^2^ and wing subunits to SEACIT^1^, with the exception of Sea3, which contributes to both entities. Due to their respective positioning in the SEAC, we refer to the Sea3^Nter^ as the Sea3^SIP^ (SEACIT portion) and the Sea3^Cter^ as the Sea3^SAP^ (SEACAT portion). A flexible linker (Sea3 connector) that tethers the wings to the core but is not visible in the density (Fig. 1c) connects the Sea3^SIP^ and Sea3^SAP^. This architecture enables the independent movement of the wings relative to the core, and suggests that in vivo, rather than two separate subcomplexes, SEACAT and SEACIT represent two modules within a single complex. This also explains why the loss of Sea3 results in detachment of SEACIT from SEACAT^15,16^.

## The SEAC is a coatomer-like complex

**Fig. 2:**
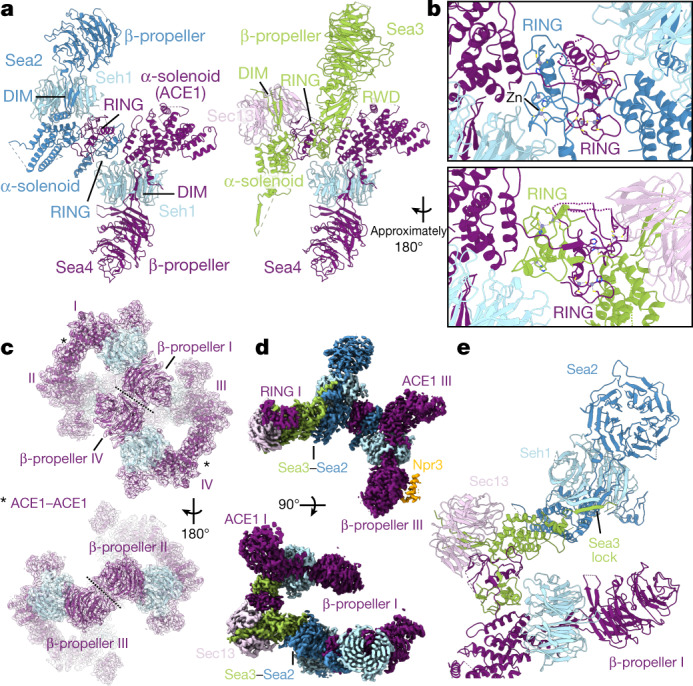
Structure of the SEAC core. **a**, Structures of Sea4–Seh1, Sea2–Seh1 and Sea3–Sec13. Both Sea2 (left) and Sea3 (right) interact with Sea4 using the C-terminal RING domain. **b**, Zoomed-in view of the RING–RING interactions between Sea2 and Sea4 (top) or Sea3 and Sea4 (bottom). **c**, Structure of the Sea4–Seh1 core cage, formed by four copies of the Sea4–Seh1 dimer. Each dimer is numbered I to IV, and interactions between copies are indicated. **d**, Protomer-focused map coloured according to the subunits showing the interaction between Sea2 and Sea3, which links opposite non-interacting copies of Sea4–Seh1. **e**, Model of the SEAC core protomer showing the Sea3 lock, an extension in the Sea3 α-solenoid that ‘locks’ the Sea2 DIM.

**Extended Data Fig. 4:**
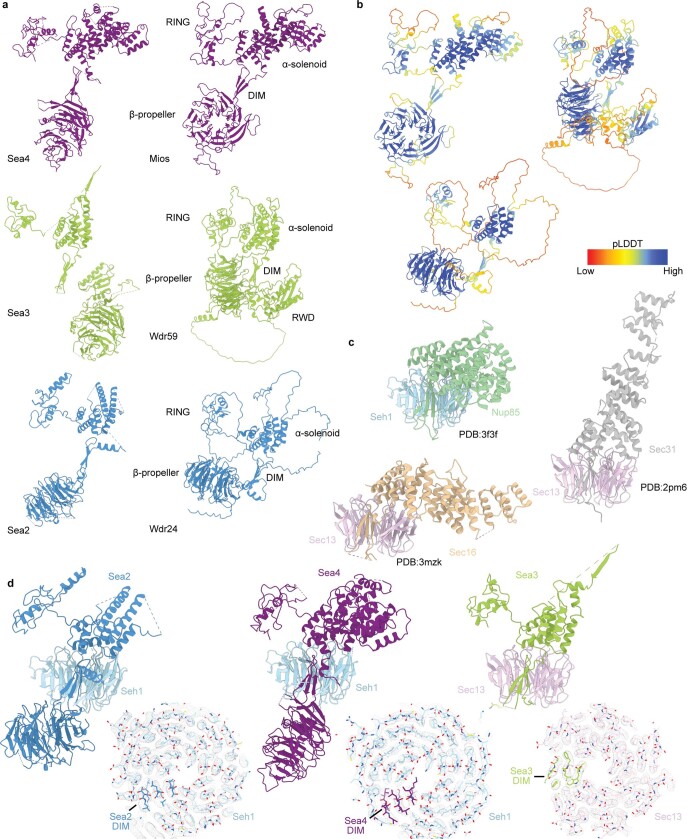
SEAC core subunits are coatomer-like proteins. **a**, Comparison of structures of Sea4, Sea3 and Sea2 with the AlphaFold predictions for their homologous GATOR2 subunits (Mios, Wdr59 and Wdr24). **b**, pLDDT score for different domains in GATOR2 subunit AlphaFold predictions. **c**, Structures of Nup85-Seh1 (PDB: 3f3f), Sec16-Sec13 (PDB: 3mzk) and Sec31-Sec13 (PDB: 2pm6). All of these subunits interact with the coatomer β-propeller through insertion of a seventh blade. **d**, Structures of Sea2-Seh1, Sea4-Seh1 and Sea3-Sec13. The domain invasion motif (DIM) of Sea2, Sea4 and Sea3 is shown with the corresponding cryo-EM density (protomer focused and Sea2-Sea3 focused maps). For Sea3, the N-terminal β-propeller was omitted for clarity.

The three main core subunits, Sea2, Sea3 and Sea4, share the same domain architecture (Fig. 1d). They consist of an N-terminal β-propeller domain, followed by a short domain-invasion motif (DIM)^17^, an α-solenoid domain and a C-terminal RING domain (Fig. 2a). This domain arrangement resembles that of coatomer proteins, arguing that these subunits evolved from a common proto-coatomer ancestor^8,18^. As expected^8^, AlphaFold predictions for the corresponding GATOR2 subunits show a similar domain organization (Extended Data Fig. 4a,b). Each of these subunits form dimers with one of the two small coatomer β-propellers present in the SEAC; specifically, Sea4 and Sea2 with Seh1, and Sea3 with Sec13 (Fig. 2a). Dimerization occurs via a mechanism similar to that observed in the nuclear pore^17^ and COPII^19^ with insertion of a DIM that contributes a seventh blade and closes the open β-propeller of Seh1 and Sec13 (Extended Data Fig. 4c,d). This locks the orientation of the N-terminal β-propeller domain with respect to the α-solenoid, fixing the angle of both domains to promote specific protein–protein interactions. As such, Seh1 and Sec13 (and β-propeller domains in general—there are 16 in the SEAC) play key structural roles, explaining their importance for SEACAT function in vivo^2^.

**Extended Data Fig. 5:**
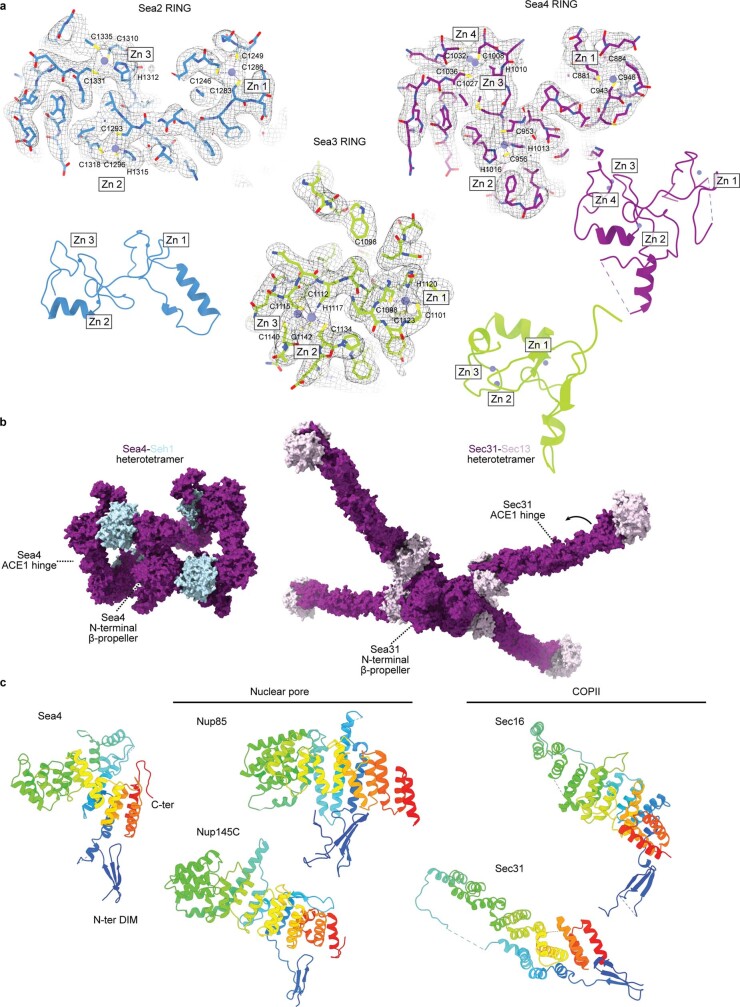
Structure of the Sea2, Sea3 and Sea4 RING domains, and formation of the SEAC cage. **a**, Structures of the Sea2, Sea4 and Sea3 RING domains are shown with the cryo-EM densities. Zn ions are numbered in order from N-terminal to C-terminal. **b**, Comparison of the SEAC cage formed by a heterotetramer of Sea4-Seh1 and the COPII cage, formed by a heterotetramer of Sec31-Sec13. The model for the COPII cage was made using PDB codes 2pm6 and 6zg6. **c**, Structure of the Sea4 ACE1 domain, and its comparison with ACE1 domains in the nuclear pore (Nup85, PDB:3f3f; Nup145C, PDB:3iko) and COPII (Sec16, PDB:3mzk; Sec31, PDB:2pm6).

Sea2–Seh1 and Sea3–Sec13 dimers each interact with one copy of Sea4–Seh1 via heterodimerization of their zinc-containing RING domains, explaining why Sea4 is present in a 2:1 ratio compared to Sea2 and Sea3 (Fig. 2b). The structures of the Sea2 and Sea4 RING domains are more similar compared to the Sea3 RING (Extended Data Fig. 5a). The RING heterodimers end up sandwiched between two coatomer β-propellers, Seh1–Seh1 (Sea2 and Sea4 RINGs) and Seh1–Sec13 (Sea3 and Sea4 RINGs). This architecture explains why removal of the RING domain of Sea2, Sea3 or Sea4 results in complex dissociation^15^. RING domains have been typically associated with E3-ubiquitin ligases^20^ and the RWD domain, present in the Sea3^SIP^, is structurally related to E2-conjugating enzymes^21^. In the SEAC, they clearly play a structural role—a ubiquitin-related function remains to be determined.

The central cage of the core is formed by a heterotetramer of Sea4–Seh1 dimers (I to IV) (Fig. 2c), in an analogous fashion to the COPII cage, where the minimal cage is formed by a heterotetramer of Sec31–Sec13 dimers^22^ (Extended Data Fig. 5b). The Sea4–Seh1 cage forms a lemniscate shape via two interfaces in the Sea4 β-propeller: copy I and IV form one flexible interaction at the front, whereas II and III close the back of the cage and interact with the wings (see below). Adjacent copies of Sea4 homodimerize via their α-solenoid domain, which folds into an ‘ancestral coatomer element 1’ (ACE1) (Extended Data Fig. 5c), a J-shaped structural motif observed in proteins from the nuclear pore and vesicle coats^23^ that has been shown to mediate homo- or heterodimerization^24^.

The Sea4–Seh1 cage is stabilized by interactions between Sea2 and Sea3, which pack their α-solenoid domains against each other and link non-interacting Sea4–Seh1 copies (I–III and II–IV) (Fig. 2d). In addition, an extension in the Sea3 α-solenoid domain (Sea3 lock) wraps around Sea2, adding a β-sheet to Seh1 and ‘locking’ the Sea2 DIM into place (Fig. 2e). Altogether, this intricate set of interactions shows that all core subunits are required to form a stable, functional complex.

## Structure of the SEAC wing

**Fig. 3:**
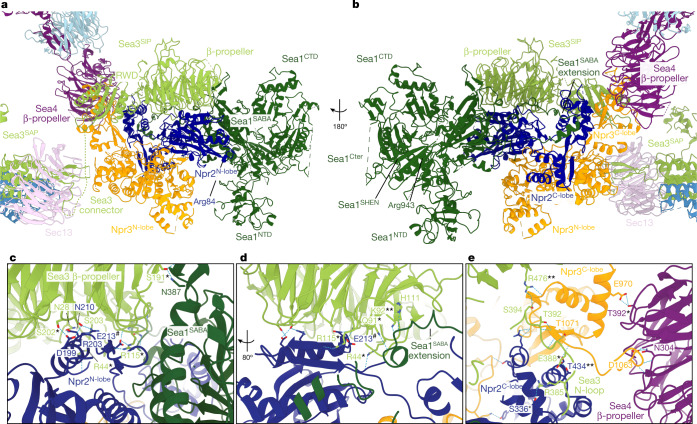
Structure of the SEAC wing. **a**,**b**, Structure of the SEAC wing (**a**), and rotated through 180° (**b**). Core elements at the interface are also shown. The positions of the two functionally important arginine residues are indicated. **c**–**e**, Interactions between the Sea3^SIP^ and Sea1–Npr2 (**c**), with view rotated by 80° (**d**) and Npr2–Npr3 (**e**). Residues in Sea3, Sea4, Npr2, Npr3 or Sea1 that are conserved in their GATOR counterparts are indicated with an asterisk, chemically similar amino acids are indicated with two asterisks and reciprocal charge reversal with a hashtag (in the case of Npr2^E213^).

**Extended Data Fig. 6:**
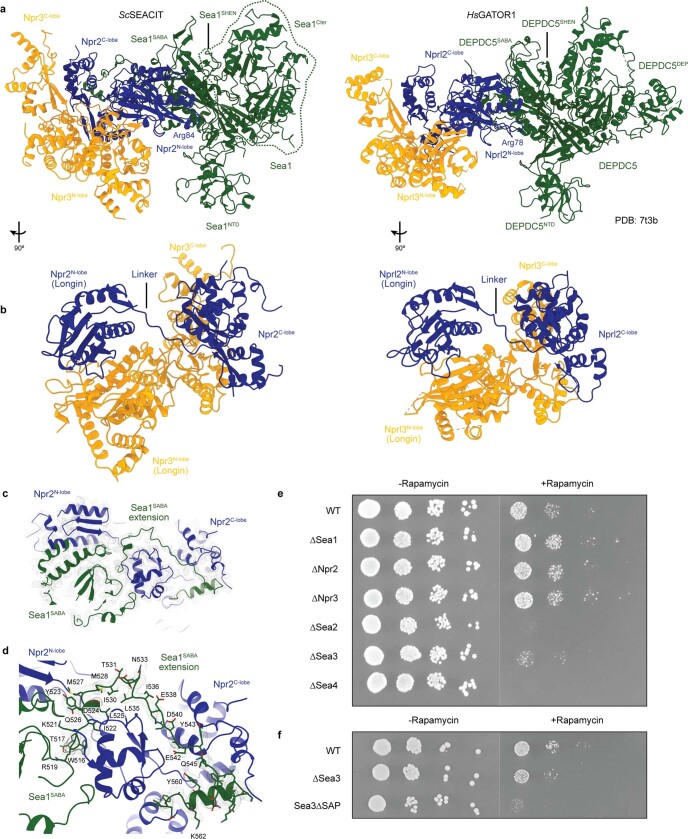
Structural conservation between SEACIT and GATOR1. **a**, Comparison between the trimeric Sea1-Npr2-Npr3 complex (SEACIT) and GATOR1 (PDB:7t3b). **b**, Comparison of the Npr2-Npr3 heterodimer (left) with Nprl2-Nprl3 (right). **c**, **d**, Sea1 interacts with Npr2 through its SABA domain, using an extension to interact with the C-lobe. **e**, Rapamycin growth assays (5 nM) of SEACAT and SEACIT deletion strains. Deletions of SEACIT subunits cause a slight increase in rapamycin resistance, whereas deletions of *SEA2* and *SEA4* cause hypersensitivity to rapamycin. *SEA3* deletion causes an intermediate SEACIT-SEACAT phenotype. **e**, Removal of the Sea3^SAP^ is sufficient to induce a SEACAT-like phenotype.

Most of the mass of the SEAC wing is formed by SEACIT subunits, Sea1, Npr2 and Npr3, which use similar interaction interfaces as GATOR1 (ref. ^25^) (Fig. 3a and Extended Data Fig. 6a). Npr2 and Npr3 consist of an N-terminal lobe (N-lobe) and a C-terminal lobe (C-lobe), which interact with each other and are connected via a linker (Extended Data Fig. 6b). The N-lobes contain the longin domains and are far from the SEAC core, whereas the C-lobe heterodimer faces the Sea4 β-propeller domain (II or III) (Fig. 3b). As detailed below, the Npr2^N-lobe^ also contains the catalytic arginine (Npr2^Arg84^) (Fig. 3a).

Sea1 is positioned at the edge of the wing and uses its SABA domain to interact with Npr2 (Extended Data Fig. 6c), whereas the C-terminal region (Sea1^Cter^), comprising the SHEN, the DEP (which is not resolved) and the CTD, is more flexible and does not interact with the rest of the wing (Fig. 3b and Extended Data Fig. 6a). We could resolve an extension in the Sea1^SABA^ that becomes partially ordered and inserts between the Sea3^SIP^ β-propeller and the Npr2^C-lobe^, interacting with and wrapping the latter (Fig. 3b and Extended Data Fig. 6d). The interaction between Sea1 and Npr2 appears to be stabilized by the Sea3^SIP^, as its β-propeller is positioned on top of the Sea1^SABA^–Npr2^N-lobe^ junction, forming a tripartite Sea1–Npr2–Sea3 interaction (Fig. 3c,d). Consistent with a role for the Sea3^SIP^ in stabilizing the wing, deletion of the *SEA3*^*SAP*^ caused cells to become hypersensitive to rapamycin similar to other SEACAT mutants, whereas deletion of *SEA3* caused a phenotype intermediate between SEACAT and SEACIT mutants (Extended Data Fig. 6e,f). Moreover, in the absence of the Sea3^SIP^, purification of SEACIT failed to yield a stable complex (data not shown). Hence, Sea3 has a dual role in both SEACIT (via the SIP) and SEACAT (via the SAP). These results are in agreement with and explain previous observations in *Schizosaccharomyces* *pombe*, where Sea3 forms part of the SEACIT^16^, and in mammals, where Wdr59 has been shown to have an inhibitory role that is required for proper mTORC1 inhibition after nutrient deprivation^26^.

**Extended Data Fig. 7:**
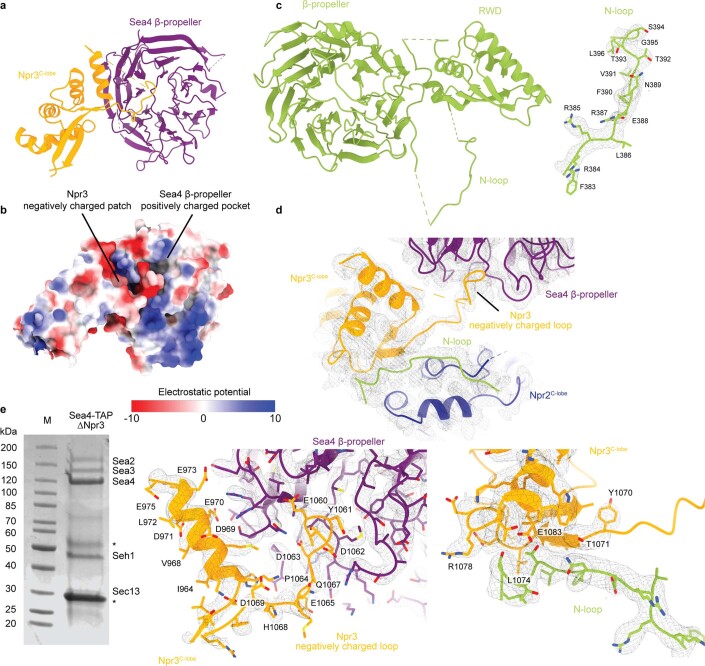
Interactions between the SEAC wing and core. **a**, Structure of the Npr3^C-lobe^ and the Sea4 β-propeller. **b**, The Npr3^C-lobe^ has a negatively charged patch that sits on top of a positively charged pocket in the Sea4 β-propeller. The electrostatic potential was calculated in ChimeraX. **c**, Structure of the Sea3^SIP^ and the N-loop. **d**, The Sea3^SIP^ N-loop interacts with both Npr2 and Npr3 C-lobes and appears to stabilize the position of the Npr3 negatively charged loop that interacts with the Sea4 β-propeller. **e**, Coomassie-stained SDS-PAGE of the SEAC purification from a Sea4-TAP ΔNpr3 strain. Only core subunits are obtained. Asterisks indicate presence of IgG and TEV protease in the eluted sample. For gel source data, see Supplementary Fig. 1.

The Npr3^C-lobe^ plays an important role in organizing the wing relative to the core, as it is sandwiched between the Sea4 β-propeller and the Sea3 RWD, in addition to contacting the Npr2^C-lobe^ (Fig. 3b). The Npr3^C-lobe^ makes the only visible interaction between the wing and the core by inserting a negatively charged loop into a positively charged pocket in the Sea4 β-propeller (Fig. 3e and Extended Data Fig. 7a,b). This interaction also appears to be stabilized by the Sea3^SIP^ via a loop (N-loop) that connects its β-propeller and RWD, which inserts on top of the Npr3^C-lobe^–Npr2^C-lobe^ interface (Fig. 3e and Extended Data Fig. 7c,d). The position and interactions of Npr3 in the wing explain why deletion of *NPR3* (ref. ^15^) or *NPRL3* (ref. ^25^) dissociates SEACIT/GATOR1 from SEACAT/GATOR2 (Extended Data Fig. 7e).

We note that despite several Sea3 residues important for interactions with Npr2, Npr3 and Sea1 being conserved in Wdr59, corresponding amino acid conservation in the Nprl2, Nprl3 and DEPDC5 partners is essentially absent (Fig. 3c–e). This potentially explains the more stable association between SEACAT and SEACIT in the SEAC, compared to GATOR1 and GATOR2 in GATOR, as well as the need for KICSTOR to stabilize GATOR in mammals^26,27^.

## The SEAC has GAP activity

**Fig. 4:**
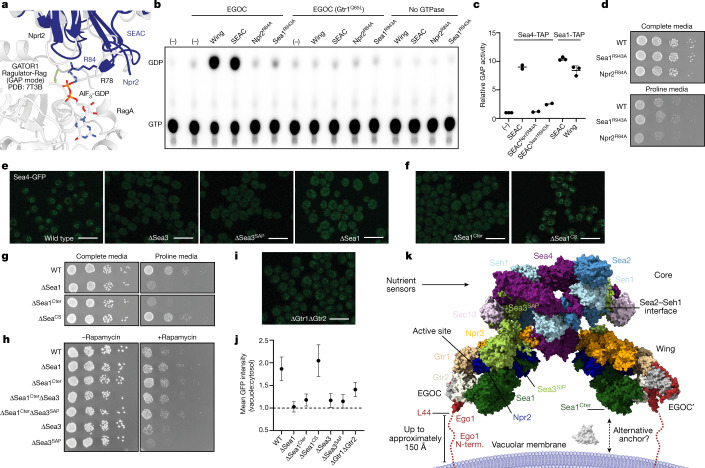
Functional analysis of the SEAC wing. **a**, Structure of the SEAC active site, superimposed on the structure of the active GATOR1-Ragulator-Rag structure. **b**, Representative thin layer chromatography of in vitro GAP assays (*n* = 2–3 independent experiments). **c**, Relative fold change in GAP activity between the wild-type SEAC, Sea1^R943A^, Npr2^R84A^ and isolated SEAC wing. The comparison between full complex and wing was done with protein purified from Sea1-TAP strains. Independent data points are presented as mean values ±s.d. where appropriate (*n* = 2 for Sea4-TAP strains and *n* = 3 for Sea1-TAP strains). **d**, Growth assays on proline wherein GAP-defective mutants present a slow-growth phenotype. **e**, SEAC localization, tracked by visualizing endogenously tagged Sea4-GFP, in wild-type (WT), ∆Sea3, ∆Sea3^SAP^ and ∆Sea1 backgrounds. **f**, SEAC localization in ∆Sea1^Cter^ and ∆Sea1^CS^ strains. **g**, Growth assays on proline for Sea1 mutant strains. **h**, Rapamycin growth assays showing that *SEA1* mutations are epistatic to *SEA3* mutations. **i**, SEAC localization in the ∆Gtr1∆Gtr2 strain. **j**, Quantification of vacuole to cytosol ratio (mean ± s.d.) of Sea4-GFP signal in cells from **e**, **f** and **i** (*n* = 30 cells per strain). **k**, Model of the SEAC bound to the EGOC in the vacuolar membrane. An alternative anchor is shown as an unknown protein that might interact with the Sea1^Cter^. Scale bars, 10 μm.

**Extended Data Fig. 8:**
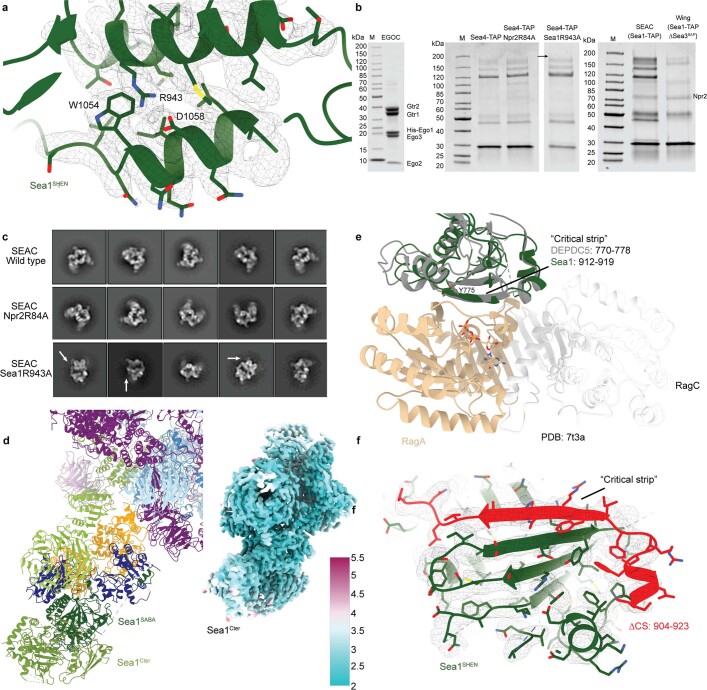
Structure of the Sea1^Cter^. **a**, Position of the Sea1^Arg943^ in the Sea1^SHEN^. **b**, Coomassie-stained SDS-PAGE of EGOC, SEAC and wing. Whereas SEAC^Npr2R84A^ shows a normal pattern of bands, SEAC^Sea1R943A^ lacks the top band that corresponds to Sea1 (indicated by an arrow). Both SEAC and wing show comparable amounts of Npr2 when purified using a TAP tag in Sea1. For gel source data, see Supplementary Fig. 1. **c**, Negative-stain 2D class averages from wild type SEAC, SEAC^Npr2R84A^ and SEAC^Sea1R943A^. White arrows indicate classes with a missing wing. **d**, Position and flexibility (showed by local resolution) of the Sea1^Cter^. **e**, Structure comparison of the Sea1^SHEN^ and DEPDC5^SHEN^ (PDB: 7t3b). **f**, Structure of the Sea1^SHEN^ showing the region deleted in the ΔSea1^CS^ strain.

Npr2^Arg84^, the GATOR1-equivalent catalytic residue located in the Npr2^N-lobe^, is solvent accessible and positioned opposite to the core–wing interface, far from any element of the core or the Sea3^SIP^ (Fig. 3a). Comparison of GATOR1-Ragulator-Rag in its active conformation^10^ with the structure of the wing shows that Npr2^Arg84^ is poised for catalysis (Fig. 4a). An arginine in Sea1 (Sea1^Arg943^), also conserved, was previously proposed to be the catalytic residue^1^. However, this residue is in a helix that is buried in the Sea1^SHEN^, and thus is unlikely to participate in catalysis (Extended Data Fig. 8a).

Our structure thus predicts that, as in GATOR1, Npr2^Arg84^ rather than Sea1^Arg943^ is the catalytic arginine and that the SEAC is in an active conformation (that is, binding of SEACAT to SEACIT is not sufficient to inhibit its GAP activity). To test these predictions, we established in vitro GAP assays using our native SEAC purifications combined with recombinantly expressed EGOC. In agreement with our structure, the SEAC was able to robustly stimulate GTP hydrolysis by Gtr1 similarly to that of isolated wing (containing the Sea3^SIP^) (Fig. 4b,c), confirming that EGOC binding is accommodated in the context of the SEAC.

To determine which arginine is responsible for catalysis, we purified native SEAC variants containing an alanine substitution in either Npr2^Arg84^ or Sea1^Arg943^ and tested them in vitro for GAP activity. Unexpectedly, both mutants were defective (Fig. 4b,c). We noted that purified SEAC^Sea1R943A^ showed a distinct pattern of bands by SDS–PAGE, where the top band corresponding to Sea1 was missing (Extended Data Fig. 8b). Thus, we further characterized these mutant complexes by negative-stain electron microscopy. Whereas two-dimensional (2D) class averages of SEAC^Npr2R84A^ showed an intact SEAC similar to wild type, 2D class averages from SEAC^Sea1R943A^ revealed the presence of abnormal complexes lacking the wing (Extended Data Fig. 8c). As Sea1^Arg943^ appears to participate in interactions with neighbouring residues (Extended Data Fig. 8a), we posit that this mutation destabilizes the structure of Sea1, which in turn destabilizes the structure of the wing, leading to reduced GAP activity.

Consistent with SEAC GAP activity being important in vivo, we observed functional defects for the Npr2^R84A^ mutation but, surprisingly, not for Sea1^R943A^ (Fig. 4d). This suggests that the Sea1^R943A^ mutation destabilizes the complex during purification, but it retains sufficient GAP activity in vivo.

## The wings tether the SEAC to the vacuole

In COPII, the Sec31–Sec13 coat is recruited to the membrane via an interaction between a flexible region in Sec31 (ref. ^28^) and the adaptor complex composed of Sec23–Sec24, which itself binds membrane-attached Sar1-GTP^29^. Like Npr2, Sec23 is also a GAP that uses an arginine finger^28^ to stimulate the GTPase activity of Sar1. Given the architectural similarities between the COPII and SEAC cages, we reasoned that a similar mechanism might occur in the SEAC, with the wing recruiting the core to the vacuolar membrane via membrane-attached EGOC.

To this end, we used an endogenous GFP tag in *SEA4* to track the localization of the SEAC in vivo. In wild-type cells, we observed strong vacuolar signal that was lost on removal of Sea3, the Sea3^SAP^ or Sea1 (Fig. 4e). This suggests that the SEAC is tethered to the vacuole via the SEACIT wings.

We tested whether the Sea1^Cter^, which is more flexible than, and lacks visible interactions with, the rest of the wing (Extended Data Fig. 8d) is specifically involved in vacuolar recruitment. Deletion of the *SEA1*^*Cter*^ not only reduced vacuolar localization (Fig. 4f), but also caused a SEACIT mutant phenotype comparable to full deletion of *SEA1* (Fig. 4g). Interestingly, deletion of the *SEA1*^*Cter*^ was epistatic to deletion of the *SEA3*^*SAP*^, highlighting that the Sea1^Cter^ plays an important role in vivo (Fig. 4h).

In the ‘inhibitory mode’, Ragulator-Rag binds to GATOR1 via the DEPDC5^SHEN^ (ref. ^25^). Albeit with low sequence conservation, the structure of the Sea1^SHEN^ is similar to the DEPDC5^SHEN^ (Extended Data Fig. 8e). As the Sea1^SHEN^ is part of the Sea1^Cter^, we tested whether the Sea1^SHEN^ is responsible for the phenotype observed in the ∆Sea1^Cter^ strain by removing the so-called ‘critical strip’ (CS; Extended Data Fig. 8f), which in GATOR1 mediates binding to RagA in the ‘inhibitory mode’^25^. We observed neither localization nor functional defects for this mutant (Fig. 4f,g), suggesting that the ‘inhibitory mode’ is not conserved in yeast, as previously proposed^9^.

The above results indicated that the SEAC is recruited to the vacuole via an interaction with the EGOC in the ‘GAP mode’. To test this hypothesis, we determined whether the deletion of *GTR1* and *GTR2*, which should remove the interaction with the EGOC, affects SEAC localization. Curiously, deleting *GTR1* and *GTR2* did not reduce vacuolar SEAC localization to the same extent as deleting Sea1 and Sea3 (Fig. 4i,j), suggesting that an additional EGOC-independent mechanism for vacuolar recruitment exists.

## Model of the SEAC on the vacuole

**Extended Data Fig. 9:**
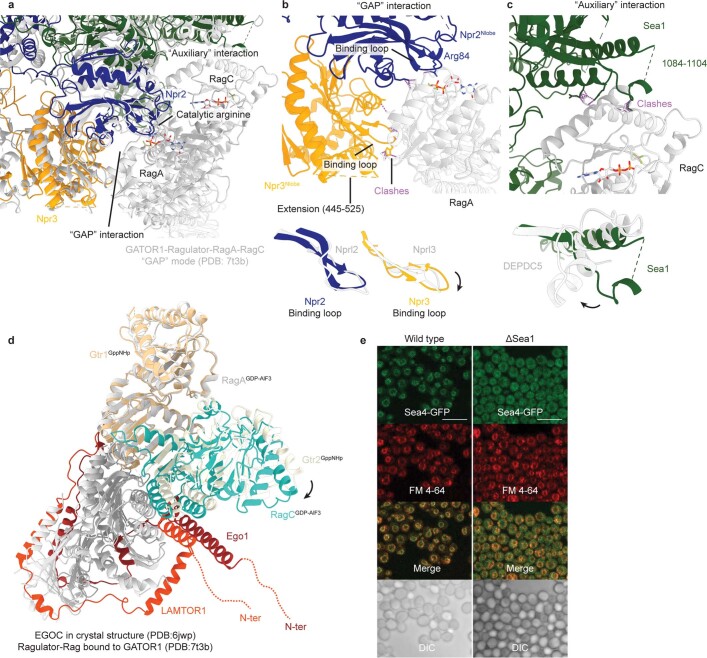
Modeling of the SEAC-EGOC super complex. **a**, Model of Ragulator-Rag bound to GATOR1 (PDB:7t3b; GAP-mode), superimposed onto the SEAC wing. The two interaction interfaces are shown. **b**, Clashes between RagA and Npr2-Npr3 in the “GAP” interaction interface are located mainly in loops from Npr2 and Npr3. From the two determined “binding” loops, only the Npr3 binding loop shows clashes. This loop in GATOR1 is folded downwards. **c**, The clash between Sea1 and RagC in the “auxiliary” interaction interface occurs in a region from Sea1 that includes a loop that folds back in DEPDC5 when bound to RagC. **d**, Comparison of the structure of Ragulator-Rag (PDB: 7t3b) and EGOC (PDB: 6jwp), superimposed on RagA-Gtr1. The conformation of RagC differs from Gtr2 due to its interaction with GATOR1. N-terminal tails of Ego1/LAMTOR1, which are anchored to the vacuole/lysosome, are oriented in a similar direction. **e**, FM 4-64 staining was used to quantify Sea4-GFP signal intensity on the vacuole vs. in the cytosol. Exemplary WT and Sea1 cells are shown (quantification in Fig. 4j). Scale bar = 10 μm.

To better understand the regulation of the SEAC, we used our structural and functional data to model the SEAC–EGOC supercomplex on the vacuolar membrane (Fig. 4k and Extended Data Fig. 9a–d). Given that the complex is active, we modelled EGOC binding to the SEAC wing in the ‘GAP mode’ on the basis of the GATOR1-Ragulator-Rag structure^10^, and considering the spatial restraint given by the flexible N-terminal tail of Ego1. Gratifyingly, binding of EGOC is well accommodated in each wing, as the last ordered N-terminal helix of Ego1 faces towards the vacuolar membrane. This tail can extend up to approximately 150 Å, which enables sufficient space to prevent the clash of the distal end of the wing with the membrane. In addition, the flexible Sea1^Cter^ is positioned facing the membrane, whereas the core ends up parallel to the membrane and faces the cytosol, making it accessible for the binding of nutrient-dependent regulators. In this configuration, the wings could accommodate different membrane curvatures given their flexibility.

**Extended Data Fig. 10:**
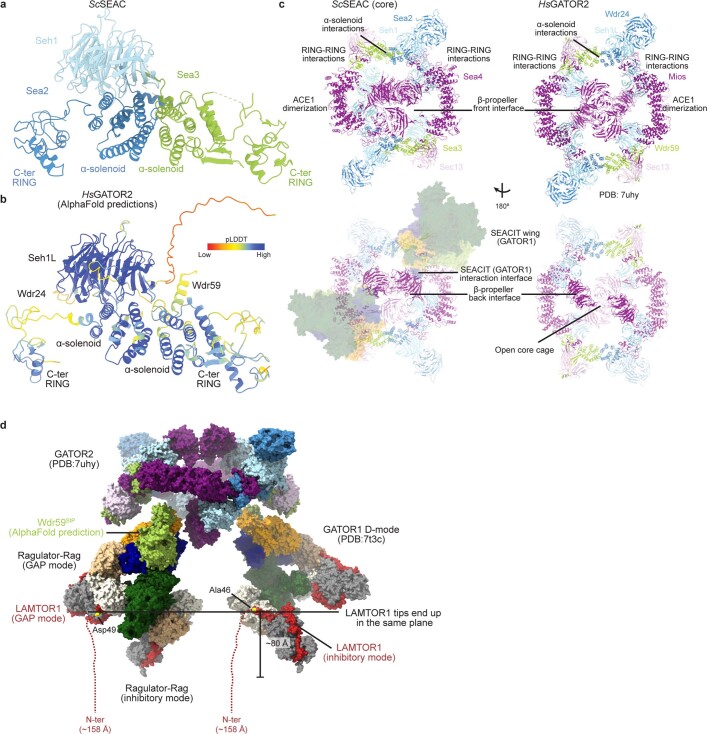
Conservation of the SEAC with GATOR2 and GATOR. **a**, Structure of the interface formed by Seh1, Sea2 and Sea3 in the SEAC. The N-terminal of Sea2 and Sea3 have been omitted for clarity. **b**, AlphaFold predictions for Seh1L, Wdr24 and Wdr59 superimposed each to their homologous SEAC subunits, colored according to the pLDDT score. **c**, Structure of the SEAC core (SEACAT) obtained in this study (left) and human GATOR2 (PDB: 7uhy, right). The main difference between the structures is the open back interface in GATOR2, which could be due to the lack of GATOR1 binding, and the visibility of the N-terminal region of Sea3 (Sea3^SIP^), which becomes ordered in the SEAC as part of the wing (not shown in the figure for clarity). **d**, Model of holoGATOR using known structures of GATOR2 (PDB: 7uhy) and an AlphaFold prediction for the N-terminal region of Wdr59, and GATOR1 bound to two copies of Rag-Ragulator, one in the “GAP mode” and one in the “inhibitory mode” (PDB: 7t3c, so-called dual mode). In the model, regardless of binding mode (GAP vs inhibitory) the first, structured residue of LAMTOR1 would be approximately equidistant to the putative position of the lysosomal membrane. GATOR2 would prevent the flipping of the wing, previously proposed to regulate GATOR1 function. Finally, DEPDC5 would be able to interact with KICSTOR. It is not known if this “dual binding mode” is physiologically relevant but appears possible given this model.

On the basis of our data and current literature, we propose that the SEAC core (that is, SEACAT) acts as a scaffolding complex that is required, but is not sufficient, to regulate the activity of the wings. Indeed, given the results of our in vitro GAP assays, a direct inhibition by the core seems unlikely. Both Sestrin2 (ref. ^30^) and CASTOR1 (ref. ^31^) bind to Wdr24 (Sea2) and Seh1L (Seh1), which in our structure form an interface next to the wings and have a conserved structure (Fig. 4k and Extended Data Fig. 10a,b). As in the mammalian system, yeast nutrient sensors may bind to the core to regulate the GAP activity of the wings (but probably require additional factors in this regulation). Collectively, our results indicate that the SEAC core (SEACAT/GATOR2) acts as a passive, rather than active, regulator of the wings (SEACIT/GATOR1).

During revision of this manuscript, the cryo-EM structure of the human GATOR2 complex was published^32^. GATOR2 has a structure virtually identical to the SEAC core (SEACAT) (Extended Data Fig. 10c) and the data are consistent with our study, further highlighting that the molecular mechanism of action of SEAC and GATOR is conserved. Modelling of a tentative GATOR holocomplex shows that GATOR2 would restrict the position of GATOR1 relative to the membrane, but would enable the binding of Ragulator-Rag in the ‘inhibitory’ and ‘GAP’ modes, potentially simultaneously (Extended Data Fig. 10d). Therefore, GATOR2 may not regulate GATOR1 through conversion between these two binding modes as previously suggested^10^.

## Methods

### Yeast strains

Yeast strains were constructed using classical recombination-based techniques. The ∆Sea1^CS^ strain was generated using CRISPR-based mutagenesis. Strains used in this study are listed in Supplementary Table 2. Plasmids are listed in Supplementary Table 3. All strains were verified by PCR and/or sequencing.

### SEAC purification

Cells expressing Sea4-TAP were grown to an optical density (OD_600_) of 4–6, collected by centrifugation at 6,000 r.p.m. for 10 min, flash-frozen in liquid nitrogen and stored at −80 °C until further use. Cells were lysed and resuspended in 3–5 volumes of lysis buffer (SEAC buffer: 20 mM HEPES–NaOH, pH 7.4, 300 mM NaCl, 5 mM CHAPS, 10% glycerol, 2 mM DTT; plus 1 mM PMSF and 1× Complete protease inhibitor tablets (Roche)). All steps were performed at 4 °C. The lysate was cleared by centrifugation at 16,000 r.p.m. (30,600 *g*) for 1 h, and the supernatant was incubated with IgG-coupled Dynabeads M270 (ThermoFisher Scientific) for 2 h. SEAC-bound beads were extensively washed with lysis and SEAC buffer then incubated overnight with TEV protease (0.1 mg ml^−1^) in elution buffer (20 mM HEPES–NaOH, pH 7.4, 300 mM NaCl, 2 mM DTT). The eluate was clarified by centrifugation at 13,000 *g* for 5 min and used immediately for cryo-EM grid preparation.

### EGOC purification

A plasmid containing Gtr1 (WT or Q65L) and Gtr2 without any affinity tag were cotransformed in *Escherichia coli* BL21* with a plasmid containing codon-optimized sequences for 6×HIS-Ego1 (∆1–37), Ego2 (∆1–7) and Ego3. Protein expression was induced by addition of 0.1 mM isopropyl-β-d-thiogalactopyranoside at 18 °C overnight.

Cells were collected by centrifugation and resuspended in lysis buffer (50 mM Tris–HCl pH 7.4, 300 mM NaCl, 5% glycerol, 20 mM imidazole, 0,15% CHAPS, 1 mM MgCl_2_, 1 μg ml^−1^ DNase, 1 μg ml^−1^ lysozyme), supplemented with 1 mM PMSF and cOmplete EDTA-free Protease Inhibitor Cocktail (Roche). Cells were lysed using an Emulsiflex system (Avestin) and cleared by centrifugation at 15,000 r.p.m. for 45 min at 4 °C. The soluble fraction was subjected to affinity purification using a chelating HiTrap FF crude column (GE Healthcare). After washing, the protein was eluted using 250 mM imidazole. Fractions containing the complex were pooled and buffer exchanged with a HiPrep 26/10 column to 25 mM HEPES–NaOH pH 7.4, 150 mM NaCl, 10% glycerol, 2 mM DTT. The sample was then applied to a MonoQ FF column (GE Healthcare) and eluted with a gradient from 150 mM to 1 M NaCl. Fractions containing the complex were incubated overnight with 20 mM EDTA at 4 °C, concentrated to 10 mg ml^−1^ (using a 100 kDa Amicon filter) and loaded on a Superdex GF200 Increase (GE Healthcare) equilibrated with 25 mM HEPES pH 7.4, 150 mM NaCl, 10% glycerol, 2 mM DTT, 2 mM EDTA. The purest fractions were collected and concentrated to 5–10 mg ml^−1^. Aliquots were flash-frozen in liquid nitrogen and stored at −80 °C until use.

### GAP assays

200 nM of EGOC (WT or Gtr1Q65L) was loaded with 40 nM [α−^32^P]GTP in loading buffer (20 mM Tris–HCl pH 8.0, 1 mM MgCl_2_, 2 mM DTT) for 30 min at room temperature. The reaction was started by mixing 1 μl wild-type, variant SEAC or wing, 4μl of loaded EGOC, 1μl of 10X GAP buffer (200 mM Tris–HCl pH 8.0, 100 mM MgCl_2_, 20 mM DTT, 20 mM GTP) and 4 μl of SEAC buffer, for a total reaction volume of 10 μl, and incubated for 30 min at room temperature. The reaction was stopped by addition of 3 μl of STOP buffer (1% SDS, 25 mM EDTA, 5 mM GTP and 5 mM GDP) and heating for 4 minutes at 65 ^o^C. The reaction (2–4 μl) was spotted on PEI Cellulose F plates (Merck) and resolved by thin layer chromatography in 1 M acetic acid and 0.8 M LiCl. After drying, the plate was exposed to a phosphor screen overnight, and imaged in a GE Typhoon phosphor imager. Quantification was performed in ImageJ 1.52p. Data were visualized and plotted in GraphPad Prism 8.

### Growth assays

Indicated strains were grown overnight in complete synthetic medium (CSM; 2% glucose, yeast nitrogen base without amino acids with ammonium sulfate, and drop-out mix). Before experiments, strains were diluted and then grown to an OD_600_ of approximately 0.1. Each strain was spotted onto CSM plates containing 0 or 5 nM rapamycin and incubated at 30 °C for 2 days before imaging. All spot assays were performed at least three times for each strain.

We observed only a weak phenotype for SEACIT mutants when grown on rapamycin (slight increase in rapamycin resistance). Because the SEAC signals nitrogen in yeast, we probed SEACIT mutants for growth on proline, a poor nitrogen source. This gave a stronger phenotype and was hence used to characterize SEACIT mutants.

For growth on proline plates, strains were made prototrophic by transformation with the pJU450 plasmid^33^ (containing *TRP1*, *HIS3* and *LEU2*). Strains were diluted in proline media to an OD_600_ of approximately 0.1 and spotted onto CSM (-Trp, -His, -Leu) plates (plus or minus proline), and incubated at 30 °C for 1–2 days before imaging. All spot assays were performed at least three times for each strain.

### Fluorescence microscopy

Strains were precultured overnight in CSM. Before imaging, strains were diluted to OD_600_ of about 0.2–0.4 and imaged at OD_600_ of about 0.7–1. Stacks of images (at least three per strain) were collected on a Zeiss LSM800 confocal laser scanning microscope and processed using ImageJ 1.52p. For comparison between strains, *Z* stacks of approximately 2 μm were averaged using maximum projection.

For quantification of the GFP signal on the vacuole, cells were grown to late exponential phase in CSM (OD_600_ approximately 0.8–1) and incubated with 5 μM FM 4-64 (ThermoFisher) for 30-40 min at 30 °C. The ratio of mean GFP signal in the vacuole versus the cytosol was calculated in ImageJ 1.52p as follows. First, a maximum projection image for the FM 4-64 channel was used to delineate the vacuole using the elliptical tool (vacuole region of interest, vROI). Then, vROI were overlaid on the summed stack DIC channel to manually trace a ROI in the cytosol (cROI) of cells with vROI using the free-hand tool, carefully trying not to include any signal from the vacuole. Only cells with a clearly stained vacuole and sufficient cytosolic area were included in the analysis (*n* = 30 cells per strain). The ratio of vacuole to cytosol GFP intensity was then calculated by measuring the mean intensity in each ROI on the maximum projection image of the GFP channel, and dividing the mean GFP intensity in the vROI by the mean GFP intensity in the cROI. This protocol ensures an unbiased way of quantification as the GFP channel is not used for any ROI determination. Data were visualized and plotted in GraphPad Prism 8.

### Sample preparation for electron microscopy

A 5 μl sample of freshly purified SEAC was applied onto QuantiFoil Au 1.2/1.3 grids previously coated with a thin layer of graphene oxide, and plunge-frozen using a Leica GP2 system at 10 °C and 90% humidity.

Negative-stain grids were prepared by applying 6 μl of sample directly onto Carbon Square Mesh, Cu, 300 Mesh, UL grids (Electron Microscopy Sciences) and incubated for 3 min. Excess sample was removed with filter paper, and grids were stained with 1% uranyl acetate, followed by removal of excess stain with filter paper and drying.

### Electron microscopy data acquisition

Cryo-EM data were acquired with EPU v.2.14 in a 300 kV Titan Krios equipped with a Falcon 4 direct electron detector and Selectris X energy filter (ThermoFisher) (DCI Lausanne). Movies were recorded in EER mode, with a total dose of 40 e/Å^2^ and target defocus range from −1.6 to −0.6 μm, slit width of 10 eV and a pixel size of 0.726 Å (×165,000 magnification). A total of 15,922 movies (in two sessions of 11,174 and 4,748 movies) were collected untilted, and 3,182 movies were collected at 35° tilt using similar microscopy settings.

Negative-stain data were acquired with EPU v.2.14 in a 120 kV Talos L120C equipped with a CETA camera (ThermoFisher) (DCI Geneva). Images were acquired at a nominal pixel size of 1.927 Å. A total of 334 images were taken for wild-type SEAC, 333 images for SEAC^Npr2R84A^ and 301 images for SEAC^Sea1R943A^.

### Cryo-EM data processing

All processing was performed in CryoSPARC v.3.2.234 (ref. ^34^). Videos were processed on-the-fly with CryoSPARC Live v.3.2.2, fractionating the movies in 40 frames, and using patch motion correction and patch CTF estimation. Resolution estimates correspond to FSC values at 0.143 using the optimized mask determined automatically after refinements, which are shown in Extended Data Fig. 2.

The data processing pipeline is detailed in Extended Data Fig. 1. Tilted and untilted datasets were preprocessed individually and merged after initial particle cleaning was performed through rounds of heterogeneous refinement and 2D classification. Particles were binned 2× (1.452 Å per pixel). A subset of micrographs from the largest untilted dataset was used to obtain an ab initio model to use as the reference volume for heterogenous refinement. All heterogenous refinement steps were performed applying *C*2 symmetry. The two smaller datasets (tilted and untilted) were merged, classified using heterogenous refinement and combined with the particles obtained from the largest untilted dataset. These combined particles were then refined using non-uniform (NU) refinement^35^, followed by symmetry expansion (*C*2), particle subtraction and local refinement using a mask on the wing. To remove partial complexes, a round of focused heterogenous refinement, using the wing volume as the reference and without imposing symmetry was performed using the non-expanded particles. This yielded one class (208,379 particles) containing intact complexes, which was unbinned to the nominal pixel size (0.726 Å per pixel) and refined using NU refinement, and local and global CTF refinement. This yielded a map at 2.95 Å resolution (consensus map), where the wings were not resolved. To improve the resolution of the wings, particles were symmetry expanded (*C*2), after which particle subtraction and local refinement was performed. Particles were further classified using three-dimensional (3D) classification without alignment, and good classes with density for the wings were merged and refined using NU refinement and local CTF refinement. This yielded a map at 2.7 Å resolution (wing focused map).

3D variability analysis^36^ revealed that the main movement of the core was due to movement of the wings, which pulled and opened the front of the cage via Sea3. Hence, we used a mask encompassing Sea2–Seh1, Sea3–Sec13, Sea4–Seh1 (defined as protomer) and performed local refinement in this region, yielding a map at 2.81 Å resolution (protomer focused map). Because the β-propeller of Sea2 is flexible outwards from the cage, it was still less resolved than the rest of the protomer. Hence, we used a smaller mask encompassing Sea2–Seh1, Sea3–Sec13, as well as the interacting Sea4 RING domains. This improved the resolution of the N-terminal region of Sea2 and its interacting copy of Seh1, yielding a map at 2.79 Å resolution (Sea2–Sea3 focused map).

All maps were sharpened using DeepEMhancer^37^.

### Negative-stain data processing

Data were processed using RELION v.4.0 (ref. ^38^). Particle picking was performed automatically using Laplacian-of-Gaussian blob detection. Selected particles were subjected to several rounds of 2D classification until a stable number of particles was obtained in the good classes.

### Model building

Structure predictions for Sea2, Sea3, Sea4, Sea1, Npr2 and Npr3 were downloaded from the AlphaFold data base^14^ (https://alphafold.ebi.ac.uk/). Regions with a very low pLDDT score (<50) were removed from the models, and folded domains with a high pLDDT score (>70) were separated and fitted to the consensus map individually in UCSF Chimera v.1.15 (ref. ^39^). There was a high correlation between the confidence score and the cryo-EM density, where most parts with a very low pLDDT score were not visible. Whereas folded domains had a good fit to the density, the major differences between our maps and the predicted models were in the orientation between the folded domains, as most of them were connected by regions with very low or low pLDDT score. Some regions that were predicted disordered were built de novo. Crystal structures of Seh1 (PDB 3F3F)^40^ and Sec13 (PDB 3MZK)^24^ were individually fitted to the density.

Model building was performed in Coot v.0.9.8.1 (ref. ^41^) and real-space refinement in Phenix v.1.20.1-4487 (ref. ^42^). First, a model for the protomer was built using the consensus, protomer-focused and Sea2–Sea3-focused maps. This model also included the region of the Npr3^C-lobe^ that interacts with Sea4. Iterative rounds of model building and real-space refinement (against the protomer-focused map) were performed until no improvement in the model was obtained. The model for the protomer was then copied and translated to fit the other copy in the consensus map in UCSF Chimera v.1.15. The core dimer was then real-space-refined against the consensus map. Wings were built individually and real-space-refined iteratively using the wing-focused map.

To build the final model, residues 961–975 of Npr3 were used to anchor the wings to the core. This region forms an α-helical stretch that is better resolved in the protomer-focused map. Because the wings are flexible relative to the core, the final model represents a tentative position based on the orientation of the Npr3^C-lobe^ in the consensus map. A final refinement was performed with the full model against the consensus map with reference model restraints to account for the lack of density for most of the wing.

The quality of the model and fit to the density was determined using MolProbity^43^ and Phenix v.1.20.1-4487 (ref. ^44^).

Figures were made in UCSF ChimeraX v.1.3 (ref. ^45^).

### Modelling of the SEAC–EGOC supercomplex

To model the SEAC–EGOC supercomplex on the vacuolar membrane, the structure of the active GATOR1-Ragulator-Rag (PDB 7T3B) was used to model EGOC binding to the wings (Extended Data Fig. 9a). The Npr3^N-lobe^ (1–590) was aligned to the Nprl3^N-lobe^ (1–174) in UCSF ChimeraX, as this alignment minimized the clash between the equivalent of the ‘binding’ loop in Nprl3 (Npr3 18–23) and RagA, while maintaining the alignment of the Npr2^N-lobe^ and Npr2^Arg84^. In GATOR1, the Nprl3 binding loop is slightly folded down compared to the Npr3 equivalent (Extended Data Fig. 9b). Importantly, a large extension in the Npr3^N-lobe^ is not positioned towards RagA, and thus can be accommodated in the context of EGOC binding. In addition, the other major clash occurs between RagC and Sea1 (residues 1090, 1400 and 1404), including a loop that is extended in our structure but folds up in GATOR1 (Extended Data Fig. 9c). Both clashes can be easily relieved by changes in the conformation of the mentioned loops/side chains without affecting the overall structure.

In the GATOR1-Ragulator-Rag structure, the conformation of the RagA–RagC heterodimer is slightly different from Gtr1^GppNHp^–Gtr2^GppNHp^ in the EGOC (PDB 6JWP)^46^ (Extended Data Fig. 9d). As such, there are more clashes with Sea1 when using this structure to model EGOC binding to the SEAC wing, using RagA as an anchor point. This is consistent with an induced-fit mechanism where Sea1 binding to Gtr2 opens the space between G-domains^10^. Likewise, severe clashes are observed when using the ‘active’ Gtr1^GTP^–Gtr2^GDP^ combination (PDB 4ARZ)^47^. Considering this, the model shown in Fig. 4 corresponds to the structure of the EGOC fitted to Ragulator-Rag using RagA and Gtr1 for the alignment. Although this model has more clashes between Sea1 and Gtr2 (compared to Sea1 and RagC), the slight shift in the EGOC would not change the orientation of the last visible N-terminal helix of Ego1, which remains facing towards the vacuolar membrane. In the model with Ragulator-Rag, the equivalent helix in LAMTOR1 also adopts the same orientation (Extended Data Fig. 9d).

### Statistics and reproducibility

No statistical methods were used to predetermine sample size, and no blinding or randomization was used. Confocal microscopy images shown in Fig. 4 are representative of at least three images of three independent experiments performed on different days with biological replicates. Confocal microscopy images shown in Extended Data Fig. 9e are representative of at least three images of two independent experiments performed on different days with biological replicates. SDS–PAGE images shown in Extended Data Figs. 1a,b, 7e and 8b are representative of at least two separate independent purifications for each. Cryo-EM micrographs shown in Extended Data Fig. 1b are representative of 15,922 (untilted) and 3,182 (tilted) movies.

### Reporting summary

Further information on research design is available in the Nature Research Reporting Summary linked to this article.

## Online content

Any methods, additional references, Nature Research reporting summaries, source data, extended data, supplementary information, acknowledgements, peer review information; details of author contributions and competing interests; and statements of data and code availability are available at 10.1038/s41586-022-05370-0.

## Supplementary information

Supplementary Information Supplementary Fig. 1 (gel source images), Table 1 (cryo-EM data collection, refinement and validation statistics), Table 2 (yeast strains used in this study) and Table 3 (plasmids used in this study).

Reporting Summary

## Data Availability

The deepEMhancer-sharpened and associated maps have been deposited in the Electron Microscopy Data Bank under the following accession codes: EMD-15364 (consensus, sharpened with a tight and wide mask), EMD-15381 (SEAC wing), EMD-15373 (protomer focused) and EMD-15374 (Sea2–Sea3 focused). The models for the SEAC and the SEAC wing have been deposited in the Protein Data Bank with accession codes 8ADL and 8AE6, respectively.
