# Supplementary material for: Cryo-EM structure of the SEA complex
Source: Nature. 2022 Oct 26;611(7935):399–404. doi: 10.1038/s41586-022-05370-0 (PMC9646525; doi:10.1038/s41586-022-05370-0)
Supplement: Supplementary file 1 — Supplementary Fig. 1 (gel source images), Table 1 (cryo-EM data collection, refinement and validation statistics), Table 2 (yeast strains used in this study) and Table 3 (plasmids used in this study). [file 41586_2022_5370_MOESM1_ESM.pdf]

nature portfolio

https://doi.org/10.1038/s41586-022-05370-0

Supplementary information

Cryo-EM structure of the SEA complex

In the format provided by the authors and unedited

Extended Data Fig. 1a

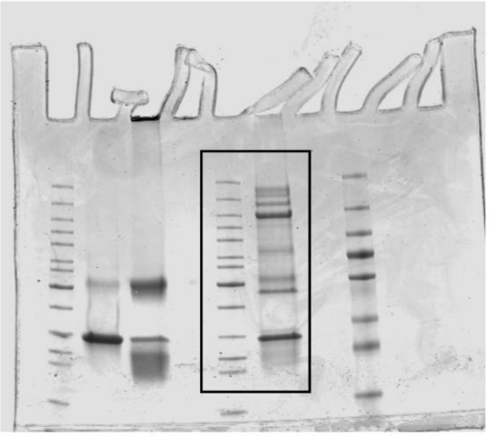

Extended Data Fig. 7e

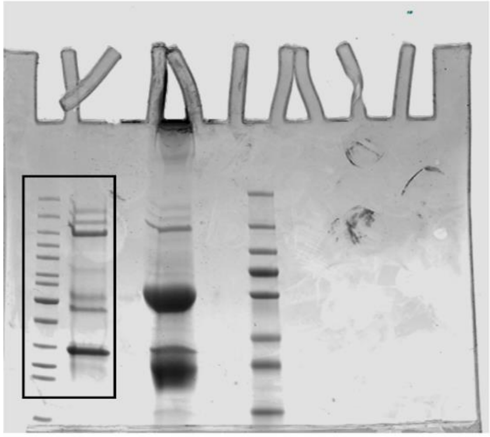

Extended Data Fig. 8b

EGOC

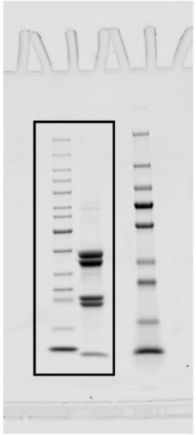

Sea4-TAP WT & Sea4-TAP Npr2^{R84A}

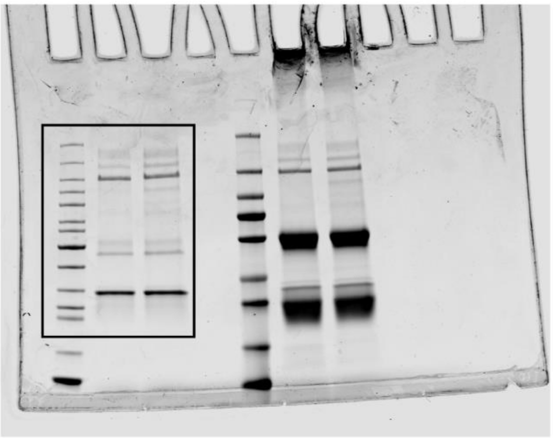

Sea4-TAP Sea1^{R943A}

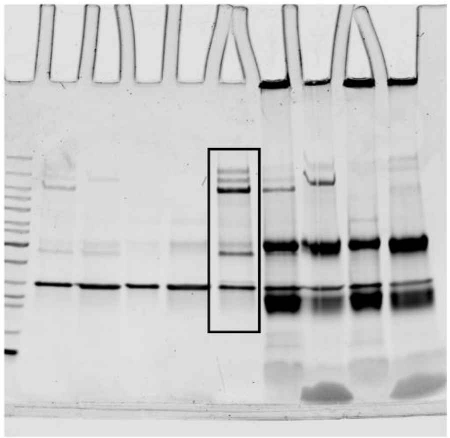

Sea1-TAP WT & Sea1-TAPΔSea3^{SAP}

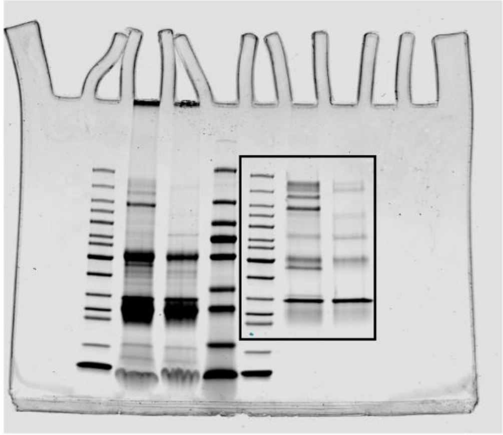

Supplementary Fig. 1. Gel source images.

	SEAC<br>(EMD-15364)<br>(PDB 8ADL)	SEAC wing<br>(EMD-15381)<br>(PDB 8AE6)	Protomer focused map<br>(EMD-15373)	Sea2-Sea3 focused map<br>(EMD-15374)
**Data collection and processing**				
Magnification	165,000			
Voltage (kV)	300			
Electron exposure ($e^{–}/Å^2$)	40			
Defocus range (μm)	-1.6 to -0.6			
Pixel size (Å)	0.726			
Symmetry imposed	C2			
Initial particle images (no.)	1,356,862			
Final particle images (no.)	208,379	416,670 (symmetry-expanded)	416,670 (symmetry-expanded)	416,670 (symmetry-expanded)
Map resolution (Å)	2.95	2.70	2.81	2.79
FSC threshold	0.143	0.143	0.143	0.143
Map resolution range (Å)	1.58-36.64	1.62-37.45	1.61-37.49	1.68-35.64
**Refinement**				
Initial model used	3f3f, 3mzk	-		
(PDB code)				
Model resolution (Å)	3.5	3.2		
FSC threshold	0.5	0.5		
Map sharpening *B* factor ($Å^2$)	-59.4	-62.4		
Model composition				
Non-hydrogen atoms	98,972	19,788		
Protein residues	12,238	2409		
Ligands	ZN: 28	0		
B factors ($Å^2$)	(min/max/mean)	(min/max/mean)		

Protein	32.1/487.72/172.41	19.87/196.71/71.31
Ligand	117.34/176.45/140.81	
R.m.s. deviations		
Bond lengths (Å)	0.004	0.004
Bond angles (°)	0.987	0.636
Validation		
MolProbity score	1.43	1.42
Clashscore	4.27	4.44
Poor rotamers (%)	0.00	0.00
EMRinger score*	2.07	3.08
Ramachandran plot		
Favored (%)	96.56	96.81
Allowed (%)	3.44	3.19
Disallowed (%)	0	0
Ramachandran Z-score		
Whole	-1.37	-1.38
Helix	-0.68	-0.89
Sheet	-0.53	-0.24
Loop	-1.16	-1.16

Supplementary Table 1. Cryo-EM data collection, refinement and validation statistics.

* DeepEMhancer-sharpened map with a tight mask.

**Name**	**Genotype**	**Source**
TB50a	MATa *leu2-3,112 ura3-52 rme1 trp1 his3*	Loewith lab
BY4742	*MATα his3Δ1 leu2Δ0 lys2Δ0 ura3Δ0*	PMID: 9483801
LT001	TB50a *SEA4-TAP[KlTrp1]*	This study
LT002	TB50a *SEA4-TAP[KlTrp1] NPR3::KanMX6*	This study
LT003	TB50a *SEA1-TAP[KlTrp1]*	This study
KH001	TB50a *SEA1-TAP[KlTrp1] SEA3*Δ546-1148-6xHA*[kanMX4]*	This study
CL001	BY4742 *SEA4-TAP[KanMX4] SEA1R943A*	This study
CL002	BY4742 *SEA4-TAP[KanMX4] NPR2R943A*	This study
RP001	TB50a *SEA1::KanMX6*	This study
KH002	TB50a *NPR2::hphNT1*	This study
KH003	TB50a *NPR3::NatNT2*	This study
KH004	TB50a *SEA2::hphNT1*	This study
KH005	TB50a *SEA3*::*KanMX6*	This study
KH006	TB50α *SEA4::NatNT2*	This study
LT004	TB50a *SEA1*Δ901-1585-6xHA*[hphNT1]*	This study
KH007	TB50a *SEA3*Δ546-1148-6xHA*[kanMX4]*	This study
KH008	TB50a *SEA1*Δ901-1585-6xHA*[hphNT1] SEA3::KanMX6*	This study
KH009	TB50a *SEA1*Δ901-1585-6xHA*[hphNT1] SEA3*Δ546-1148-6xHA*[kanMX4]*	This study
4077	TB50α *SEA1Δ904-923::20GS*	This study
NIC070	BY4742 *SEA1R943A*	Claudio DeVirgilio
NIC071	BY4742 *NPR2R943A*	Claudio DeVirgilio
LT005	TB50a *SEA4-GFP[KlTrp1]*	This study
KH010	TB50a *SEA4-GFP[KlTrp1] SEA1::KanMX6*	This study
KH011	TB50a *SEA4-GFP[KlTrp1] SEA3::KanMX6*	This study

KH012	TB50a *SEA4-GFP[KlTrp1]* *SEA3*Δ546-1148-6xHA*[kanMX4]*	This study
KH013	TB50a *TRP::SEA4-GFP SEA1*Δ901-1585-6xHA*[hphNT1]*	This study
CL003	TB50a *SEA4-GFP[KlTrp1] SEA1Δ904-923::20GS*	This study
CL004	TB50a *SEA4-GFP[KanMX4] GTR1::TRP GTR2::hphNT1*	This study

Supplementary Table 2. Yeast strains used in this study.

**Plasmid**	**Description**	**Source**
pJU450	*pRS415; TRP1, HIS3*	PMID: 17560372
pST51-Ego2-Ego3-HIS-Ego1	*pST51; EGO2Δ1-7, EGO3, 6xHIS-EGO1Δ1-37*	This study
pST93-Gtr1-Gtr2	*pST93; GTR1,GTR2*	This study
pST93-Gtr1^{Q65L}-Gtr2^{WT}	*pST93; GTR1^{Q65L},GTR2*	This study

Supplementary Table 3. Plasmids used in this study.
