# Supplementary material for: Cryo-EM structure of the SEA complex
Source: Nature. 2022 Oct 26;611(7935):399–404. doi: 10.1038/s41586-022-05370-0 (PMC9646525; doi:10.1038/s41586-022-05370-0)
Supplement: Supplementary file 2 — Reporting Summary [file 41586_2022_5370_MOESM2_ESM.pdf]

nature portfolio

Corresponding author(s): Robbie Loewith

Last updated by author(s): Sep 5, 2022

Reporting Summary

Nature Portfolio wishes to improve the reproducibility of the work that we publish. This form provides structure for consistency and transparency in reporting. For further information on Nature Portfolio policies, see our Editorial Policies and the Editorial Policy Checklist.

Statistics

For all statistical analyses, confirm that the following items are present in the figure legend, table legend, main text, or Methods section.

n/a	Confirmed	
☐	☒	The exact sample size (*n*) for each experimental group/condition, given as a discrete number and unit of measurement
☐	☒	A statement on whether measurements were taken from distinct samples or whether the same sample was measured repeatedly
☒	☐	The statistical test(s) used AND whether they are one- or two-sided<br>*Only common tests should be described solely by name; describe more complex techniques in the Methods section.*
☒	☐	A description of all covariates tested
☒	☐	A description of any assumptions or corrections, such as tests of normality and adjustment for multiple comparisons
☐	☒	A full description of the statistical parameters including central tendency (e.g. means) or other basic estimates (e.g. regression coefficient) AND variation (e.g. standard deviation) or associated estimates of uncertainty (e.g. confidence intervals)
☒	☐	For null hypothesis testing, the test statistic (e.g. *F*, *t*, *r*) with confidence intervals, effect sizes, degrees of freedom and *P* value noted<br>*Give P values as exact values whenever suitable.*
☒	☐	For Bayesian analysis, information on the choice of priors and Markov chain Monte Carlo settings
☒	☐	For hierarchical and complex designs, identification of the appropriate level for tests and full reporting of outcomes
☒	☐	Estimates of effect sizes (e.g. Cohen's *d*, Pearson's *r*), indicating how they were calculated

Our web collection on statistics for biologists contains articles on many of the points above.

Software and code

Policy information about availability of computer code

Data collection	EPU 2.14 for microscope data collection (ThermoFischer)
Data analysis	CryoEM: CryoSPARC v3.2.234, CryoSPARC Live v3.2.2, RELION 4.0 and DeepEMhancer for data processing. UCSF Chimera 1.15 and ChimeraX 1.3 for data visualization and figure preparation, WinCoot 0.9.8.1, PHENIX 1.20.1-4487 and MolProbity for model building. ImageJ 1.52p (NIH) for fluorescence imaging processing and GAP assay quantification, GraphPad Prism 8 for visualization and plot preparation of GAP and GFP signal quantification.

For manuscripts utilizing custom algorithms or software that are central to the research but not yet described in published literature, software must be made available to editors and reviewers. We strongly encourage code deposition in a community repository (e.g. GitHub). See the Nature Portfolio guidelines for submitting code & software for further information.

Data

Policy information about availability of data

All manuscripts must include a data availability statement. This statement should provide the following information, where applicable:

- Accession codes, unique identifiers, or web links for publicly available datasets
- A description of any restrictions on data availability
- For clinical datasets or third party data, please ensure that the statement adheres to our policy

The deepEMhancer-sharpened and associated maps have been deposited in the Electron Microscopy Data Bank under the following accession codes: EMD-15364 (consensus, including a tight and wide mask), EMD-15381 (SEAC wing), EMD-15373 (protomer focused) and EMD-15374 (Sea2-Sea3 focused). The models for the SEAC and the SEAC wing have been deposited in the Protein Data Bank with accession codes 8ADL and 8AE6, respectively. For model building, the following models

from the PDB were used: 3mzk and 3f3f. AlphaFold accession numbers for SEAC subunits used for initial models are: P38164, Q03897, Q08281, P47170, P39923, P38742.

Field-specific reporting

Please select the one below that is the best fit for your research. If you are not sure, read the appropriate sections before making your selection.

☒ Life sciences ☐ Behavioural & social sciences ☐ Ecological, evolutionary & environmental sciences

For a reference copy of the document with all sections, see nature.com/documents/nr-reporting-summary-flat.pdf

Life sciences study design

All studies must disclose on these points even when the disclosure is negative.

Sample size	No sample size calculation was performed. Two cryo-EM data sets were collected on the SEAC and the number of good particles obtained after classification was sufficient for obtaining a high-resolution reconstruction. For GAP assays, the number of replicates used for the analysis was based on reproducibility from preliminary experiments. For localization analyses, the number of cells used was determined after analyzing a different number of images from a subset of experiments and observing the differences in means and SD obtained.
Data exclusions	No data collected was excluded, only some particles were discarded during cryo-EM data processing as its required during the procedure.
Replication	Spot assays were performed at least 3 times with successful replication. In vitro GAP activity assays were performed 2 to 3 times, with consistent results. Fluorescence images were collected at least 2 times for each strain in different days, with reproducible results. Electron microscopy images (negative stain and cryo-EM) were reproducibly obtained in different days with different protein purifications.
Randomization	Randomization was not performed as it is not applicable for the current study. Samples of known composition were required and used.
Blinding	No blinding was performed as there was no randomization. Samples of known composition were required and used.

Reporting for specific materials, systems and methods

We require information from authors about some types of materials, experimental systems and methods used in many studies. Here, indicate whether each material, system or method listed is relevant to your study. If you are not sure if a list item applies to your research, read the appropriate section before selecting a response.

Materials & experimental systems

n/a	Involved in the study	
☒	☐	Antibodies
☒	☐	Eukaryotic cell lines
☒	☐	Palaeontology and archaeology
☒	☐	Animals and other organisms
☒	☐	Human research participants
☒	☐	Clinical data
☒	☐	Dual use research of concern

Methods

n/a	Involved in the study	
☒	☐	ChIP-seq
☒	☐	Flow cytometry
☒	☐	MRI-based neuroimaging
